# Variation in maternal lactation practices associated with changes in diurnal maternal inflammation

**DOI:** 10.1038/s41598-024-54963-4

**Published:** 2024-02-22

**Authors:** Carmen Hove, Kristine Joy Chua, Melanie Ann Martin, Madison Hubble, Amy M. Boddy

**Affiliations:** 1 University of California, Santa Barbara, USA; 2 University of Washington, Seattle, USA

**Keywords:** Reproductive biology, Biological anthropology, Behavioural ecology

## Abstract

While the importance of human milk in shaping infant immune function is well established, the impact of at-the-nipple (ATN) breastfeeding on maternal immune status has been understudied. Since lactation evolved to support infant survival and boost maternal fitness, we predict that ATN breastfeeding will confer benefits on maternal immune function. We measure the absolute and relative frequency of different infant feeding methods (ATN breastfeeding, pumping, donated milk, other supplementation) used by postpartum women in Seattle, WA (USA). We implement Bayesian modeling to estimate the effects of ATN breastfeeding on diurnal change in secretion rate of “pro-inflammatory” salivary cytokines and C-reactive protein (CRP). Our results show that most mothers in our sample used a variety of infant feeding methods, with pumping as the most common alternative to ATN breastfeeding. We find that ATN breastfeeding is associated with non-linear effects on diurnal IL-8 and CRP. Furthermore, we find that women who report zero versus ubiquitous ATN breastfeeding exhibit opposing diurnal patterns in CRP secretion rate. This study provides evidence that variation in maternal lactation practices corresponds to differences in maternal immune responses, highlighting how measuring lactation as a continuous variable can further enhance understanding of postpartum maternal physiology.

## Introduction

### Overview

The well-documented benefits of human milk for infant immune function (e.g., reduced short-term morbidity and mortality from infectious disease)^1,2^, especially in regions without access to clean drinking water, form the basis for current public health guidance on breastfeeding. Considering lactation evolved as a key form of maternal investment crucial for both infant survival and maternal fitness, lactation may also confer benefits on maternal immune function, especially in the early postpartum period when maternal mortality risk is highest^3^. To date, however, testing this hypothesis in humans has been complicated by both a relative scarcity of data on postpartum maternal immune function and predominance of infant-centered and binary/categorical measures of breastfeeding behavior (e.g., simple presence/absence of supplementation)^4–6^. Perhaps most critically, we know of very few studies that have separated the immunological effects of at-the-nipple (ATN) breastfeeding from breastfeeding-by-pumping, an evolutionarily novel infant feeding method that preserves many, but not all, of the physiological aspects of ATN breastfeeding. To address these gaps in knowledge, we measure the absolute and relative frequency of ATN breastfeeding used by postpartum mothers during a 24-h collection period and estimate the effects of ATN breastfeeding (modeled as a continuous variable) on salivary markers of inflammation.

### Lactation and immune function

The transition from pregnancy (a phase of female reproduction requiring immunological tolerance of a semi-allogeneic fetus^7^) to the postpartum period is marked by metabolic and hormonal recalibration^8,9^ and potentially unique demands on immune competence (e.g., heightened pathogen clearance)^10^. Prior research suggests that the hormonal milieu involved in sustained lactation (e.g., elevated oxytocin and prolactin, reduced progesterone) may bolster immune competence while suppressing hyper-inflammation^11–18^. In humans, greater energetic throughput and tighter regulation of metabolic “resetting” induced by regular lactation^19,20^ may also exert anti-inflammatory effects, given the immunological benefits associated with exercise^21^, regulated caloric restriction^22^ and reduced adiposity^23^. To date, however, much of the research on potential mechanisms through which lactation may regulate inflammatory processes has been done outside the context of the postpartum period (e.g., ex vivo studies, use of animal models, exogenous oxytocin dosage in men). Of the studies that have focused specifically on postpartum women, evidence is mixed: some research reports that breastfeeding is associated with heightened and activated innate and specific immune defenses^24^ while other studies show no effect of lactation on inflammation^25^. Greater intensity of breastfeeding has been associated with reduced long-term risk of breast^26^ and ovarian cancer^27^. However, such observational studies (commonly conducted in economically developed populations) are often plagued by similar confounds (e.g., higher socioeconomic status and better health care access) as those demonstrating long-term benefits of breastfeeding for infants^28^. There is some evidence, at the proximate level, that breastfeeding activates tumor immune response, but such findings are complicated by categorical measures of breastfeeding (e.g., presence/absence of any reported breastfeeding)^29^.

### Infant feeding behavior from the maternal perspective

A growing number of women worldwide are provided with access to evolutionarily novel alternatives to at-the-nipple breastfeeding (e.g., electric breast pumps, formula) while simultaneously directed to provide only human milk to infants six months of age and younger. Current World Health Organization (WHO) guidelines recommend “exclusive breastfeeding for the [infant’s] first 6 months of life”. Similarly, the United States Centers for Disease Control (CDC) recommends a minimum of six months exclusive breastfeeding, defined as a situation in which an infant is fed “only breast milk—no solids, water, or other liquids”, even though complete lack of supplementation is rare even in natural fertility populations^30,31^.

One key feature of these guidelines is that they do not differentiate between breastfeeding at-the-nipple and lactation-by-pumping (henceforth referred to as pumping). Pumping, especially using modern pumps with customizable settings, effectively mimics the mechanical aspects of infant suckling^32^, thus encouraging a similar positive feedback loop that sustains milk production while removing direct nipple contact between mother and infant. Reduced at-the-nipple contact might alleviate risk of direct microbial transfer from infant to mother, but breast pumps themselves can easily become contaminated, presenting an alternative source of pathogen exposure^33^. Pumping is a particularly useful infant feeding method for when it may be inconvenient, difficult or impossible for a mother to engage in at-the-nipple breastfeeding. Additionally, pumping helps maintain milk supply if the infant is born prematurely and/or has problems latching and more time is needed to successfully establish breastfeeding^33,34^. On the other hand, pumping may attenuate the predicted benefits of lactation on maternal physiology by disrupting the rhythmicity of milk production and ejection elicited by on-demand ATN breastfeeding, increasing risk of milk stasis (a primary cause of breast inflammation)^35^ and removing or attenuating important signaling pathways between mother and infant (e.g., hormonal regulation, retrograde flow effects of microbiota)^36,37^. Given the growing prevalence of pumping as a feasible alternative to ATN breastfeeding^38^, it is critical to (1) develop more fine-grained measures of lactation that differentiate between these two methods and (2) use these measures to separate out the effects of ATN breastfeeding versus pumping on maternal physiology.

### Objectives and predictions

For this study, we developed a novel method for quantifying infant feeding behavior that (1) differentiated between lactation that occurred at-the-nipple versus lactation using a pump, (2) allowed women to report multiple infant feeding methods used for each feeding bout, and (3) produced continuous rather that categorical/binary measures of infant feeding behavior. We then used these data to estimate the effects of at-the-nipple (ATN) breastfeeding on evening-to-morning change in “pro-inflammatory” maternal salivary cytokines (IL-1ß, IL-6, IL-8, TNF-α) and C-reactive protein (CRP), accounting for the separate effects of pumping. Data collection occurred during the COVID-19 pandemic stay-at-home order, which provided a rare ‘natural control’ for concurrent pathogen exposure.

We chose to measure IL-1ß, IL-6, IL-8, TNF-α, and C-reactive protein (CRP) because these markers can be reliably detected in saliva, provide information on both specific inflammatory pathways (cytokines) and non-specific systemic inflammation (CRP), and should therefore provide a snapshot of current inflammatory status^39^ (Table 1, Table S1). Most salivary cytokines peak at time of waking^40–43^, with adverse conditions (e.g., sleep deprivation, PTSD, sleep apnea, obesity, arthritis, cardiovascular disease, domestic abuse) commonly associated with higher morning peaks in inflammatory cytokine levels^44–47^. Based on this knowledge, we predicted that morning peak in inflammatory markers would be comparatively attenuated among mothers reporting high incidence of ATN breastfeeding. In other words, we predicted that ATN breastfeeding would be negatively associated with evening-to-morning change in IL-1ß, IL-6, IL-8, TNF-α, and CRP secretion rate.

**Table 1:** Functional descriptions for each salivary immunological biomarker.

Measure	Description
CRP	Acute phase protein synthesized by the liver in response to cytokine stimulation during an inflammatory event; a non-specific measure of systemic inflammation.
IL-1β	Pyrogenic cytokine secreted by monocytes and macrophages. Activated by exposure to essentially all microbial products via TLR ligands. Mediates inflammatory response and immune cell activity.
IL-6	Secreted by macrophages, osteoclasts, and smooth muscle cells. Inhibits effects of TNF-α. Mediator of acute phase response. Stimulates acute phase protein synthesis, production of neutrophils, and B cell growth. Inhibits regulatory T cells.
IL-8	Chemokine produced by macrophages and epithelial cells. Exerts strong specificity for neutrophils, weak effects on other leukocytes. Stimulates phagocytosis by recruited immune cells.
TNF-α	Both a pyrogenic cytokine and an adipokine. Promotes insulin resistance. Produced by macrophages. Escalates inflammatory response.

## Results

The demographics of our sample were roughly similar to those of the general Seattle population reported in the 2022 Census (https://www.census.gov/quickfacts/fact/table/WA/PST045222): 75.00% of our participants reported a household income over $100,000 (Table S2), 55.21% reported holding a professional degree (Table S3), 89.58% were married or in a domestic partnership (Table S4), and 67.71% were of European descent (Table S5). Table S6 shows the number and percent of participants by reported employment status. The median age at time of data collection was 34 but ranged from 25 to 42 (Table 2). The median number of days since delivery was 70, ranging from 8 to 212 days (Table 2). Only one participant reported giving birth to twins, while all other participants reported a singleton birth. 27.08% of mothers reported giving birth via Cesarean section (slightly more than the 21% rate reported for Washington by the CDC in 2021) and 44.79% reported having a birth complication of some kind (e.g., pre-eclampsia, delivery 3+ weeks before due date, gestational diabetes, gestational hypertension, breech presentation, manual removal of placenta) (Table 3).

**Table 2:** Median, interquartile range, mean, standard deviation, and range values for numeric variables.

Variable	Median	IQR	Mean	Standard deviation	Min	Max
Age	34.00	3.00	34.34	3.38	25.00	42.00
Days since delivery	70.00	88.25	86.29	54.59	8.00	212.00
Total infant feedings (24 h)	10.00	4.00	11.09	3.33	5.00	20.00
Hours between samples (24 h)	10.38	2.42	12.92	7.30	5.68	34.62
% ATN breastfeeding (24 h)	91.99	35.00	74.57	34.63	0.00	100.00
% Pumping (24 h)	0.00	22.22	18.66	29.46	0.00	100.00
Pre-pregnancy BMI	24.56	7.71	26.59	5.79	18.38	45.49
Current BMI	26.61	5.28	27.65	5.47	19.30	48.92
Parity	2.00	1.00	1.90	1.02	1.00	5.00

**Table 3:** Incidence rate for binary variables.

Variable	Yes	No	Incidence (%)
Maternal gastrointestinal Illness	1	95	1.04
Maternal severe respiratory illness	1	95	1.04
Maternal minor cold	4	92	4.17
Lactational amenorrhea	11	85	11.46
Co-sleeping (24 h)	21	75	21.88
C-section	26	70	27.08
Alcohol (24 h)	30	66	31.25
Birth complication(s)	43	53	44.79
Night feeding (24 h)	81	15	84.38

### COVID-19 pandemic created unique environment

All data were collected during COVID-19 stay-at-home order. Most women (86.46%) reported at least one “adverse” effect of the pandemic, with 78.12% of participants reporting social isolation and 61.46% reporting heightened anxiety (Table 4). Conversely, our participants reported very low incidence of infectious disease symptoms (likely because of social isolation). Only 4 individuals (4.17%) reported having “a minor cold that made [them] feel uncomfortable but did not keep [them] sick in bed” at some point in the week prior to data collection, while one participant (1.04%) reported incidence of a “stomach bug due to infection (i.e., diarrhea, vomiting)” or “respiratory infection more severe than a minor cold” in the preceding week (Table 3).

**Table 4:** Incidence of reported COVID-19 effects.

COVID-19 effect	N	Percent (%)
Reduced access to mental health services	9	9.38
Loss of employment	10	10.42
Financial stress	21	21.88
Loss of childcare	24	25.00
Reduced access to postpartum support	41	42.71
Heightened anxiety	59	61.46
Social isolation	75	78.12

### Most mothers employed a mixture of infant feeding methods

During the 24-h collection period, only 11.46% (n = 11) of participants in our sample reported a complete absence of ATN breastfeeding, 38.54% (n = 37) individuals indicated exclusive ATN breastfeeding, and 50% (n = 48) indicated using a mix of ATN breastfeeding and other infant feeding methods (Table S7). When combined with reported long-term history of infant feeding behavior, only two participants in our sample had *never* breastfed their infant at-the-nipple. Both these individuals reported reliance on pumped milk, therefore no participants in our sample exhibited exclusive reliance on infant formula. Only 8.33% (n = 8) of participants reported a history of using exclusive at-the-nipple breastfeeding with no supplementation or replacement. In sum, 3.12% of participants (n = 3) reported ever using donated breast milk, 15.62% (n = 15) indicated use of solid foods, 32.29% (n = 31) reported using non-breastmilk liquids at some point, and 85.42% (n = 82) reported either current or past use of expressed/pumped milk.

### Morning rise in IL-8 highest among women who reported partial reliance on ATN breastfeeding

**Figure 1:**
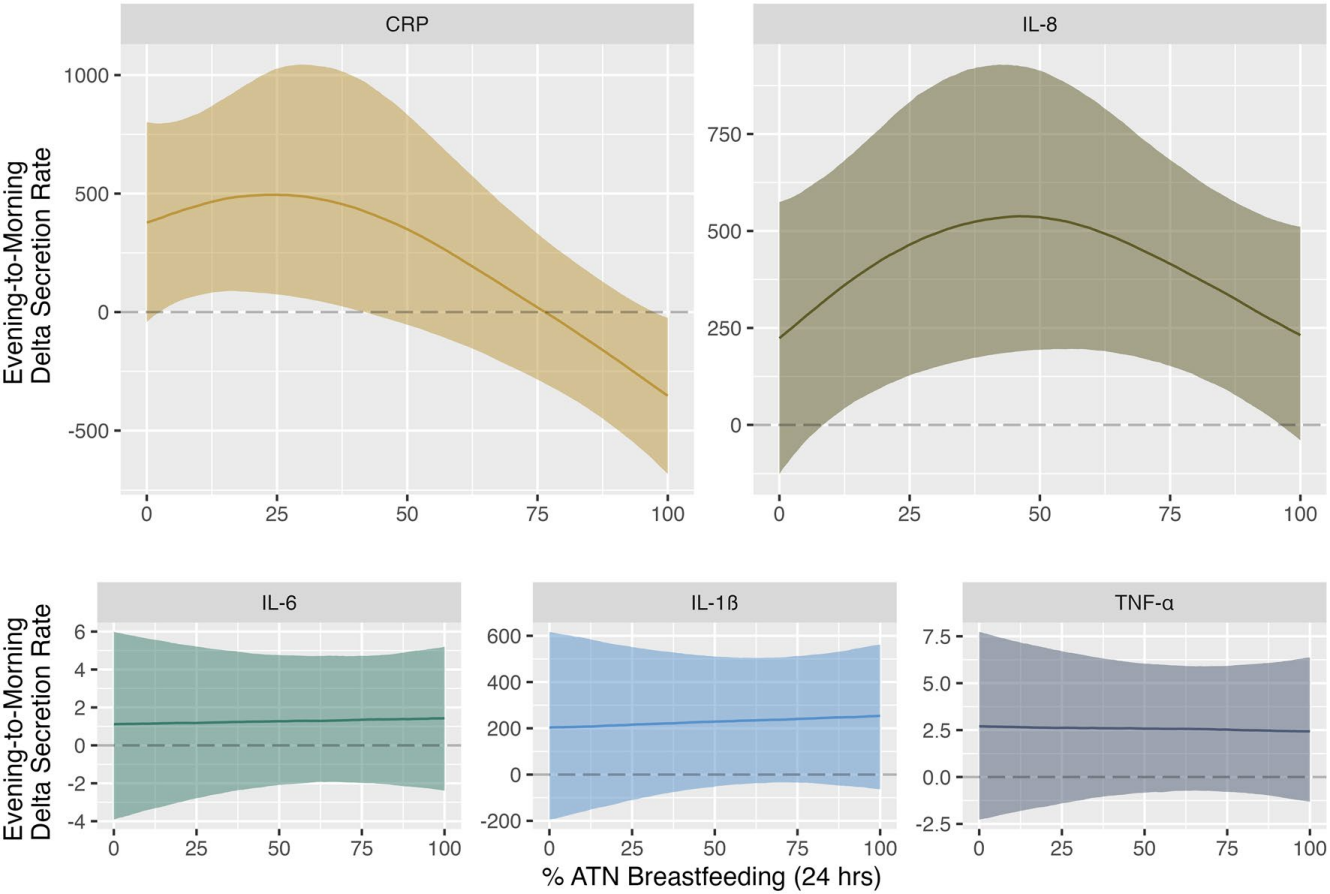
Predicted evening-to-morning change in CRP, IL-8, IL-6, IL-1ß, and TNF-α secretion rates by % ATN breastfeeding. Solid lines: point estimates for predicted median value. Shaded regions: 95% credible intervals. Dotted lines: zero difference between evening and morning secretion rate. Values above the horizontal dotted line: secretion rate is higher in the morning. Values below the horizontal dotted line: secretion rate is higher in the evening.

**Figure 2:**
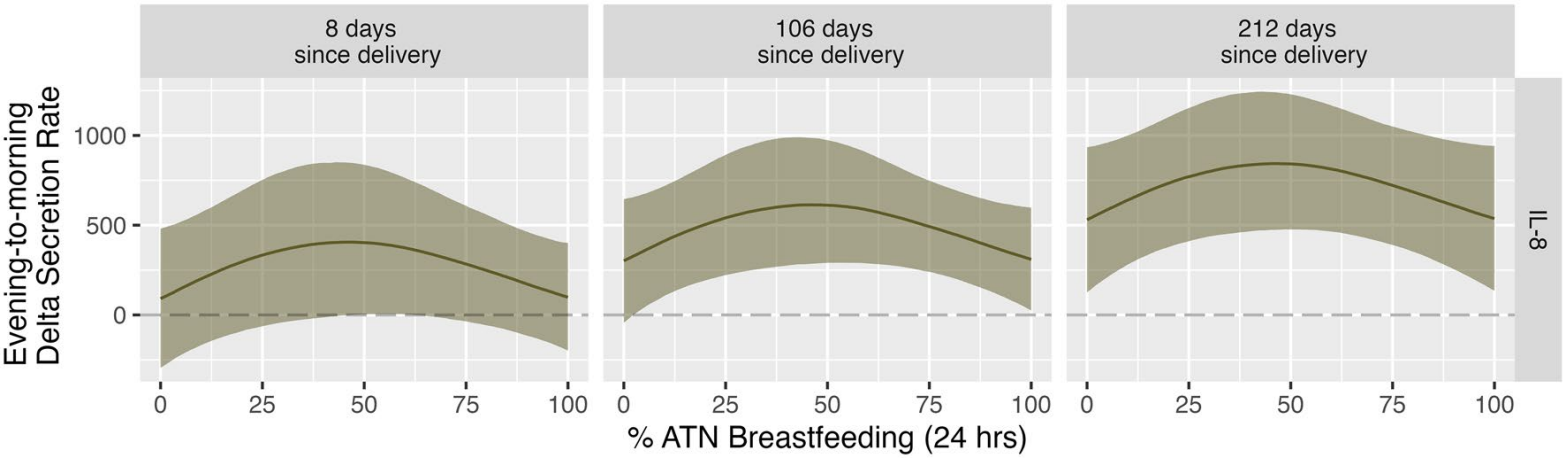
Predicted median evening-to-morning change in IL-8 by % ATN breastfeeding and time since delivery. Solid lines: point estimates for predicted median value. Shaded regions: 95% credible intervals. Dotted lines: zero difference between evening and morning secretion rate. Values above the horizontal dotted line: secretion rate is higher in the morning. Values below the horizontal dotted line: secretion rate is higher in the evening.

As shown in Table S8, median evening IL-8 secretion rate was 127.79 pg/mL/min (IQR = 216.83 pg/mL/min) while median morning IL-8 secretion rate was 269.24 pg/mL/min (IQR = 457.38 pg/mL/min). We found a strong non-linear effect of % ATN breastfeeding on evening-to-morning change (i.e., delta) in IL-8 secretion rate (Variance = 359.42; 95% CI = 25.3, 986.82) (Fig. 1). As described in Table 5, median evening-to-morning rise in IL-8 secretion rate was similar among mothers reporting lowest and highest reliance on ATN feeding, with the maximum predicted rise in IL-8 secretion rate (Median: 538.20 pg/ml/min; 95% CI: 189.85, 925.39) occurring among mothers reporting a moderate rate of 46% ATN breastfeeding. While there was no effect of % pumping on diurnal IL-8 secretion rate (Table S9, Fig. S3), time since delivery was positively associated with morning-to-evening rise in IL-8 secretion rate (Table S9, Fig. S4). Figure 2 shows the effect of increasing time since delivery on predicted values for delta IL-8 secretion rate. Conversely, % ATN breastfeeding, % pumping, and time since delivery were not associated with any robust change in evening-to-morning IL-1ß, IL-6, or TNF-α secretion rate (Table S9, Fig. 1, Figs. S3, S4).

**Table 5:** Predicted median values and associated 95% credible intervals for evening-to-morning change in CRP and IL-8 secretion rate by % ATN breastfeeding.

Measure	ATN breastfeeding (%)	Predicted value
CRP	0	Median: 377.95 pg/ml/min; 95% CI: −41.86, 801.37
CRP	25	Median: 495.16 pg/ml/min; 95% CI: 75.08, 1026.14
CRP	100	Median: −352.97 pg/ml/min; 95% CI: −682.15, −24.56
IL-8	0	Median: 223.49 pg/ml/min; 95% CI: −125.97, 574.30
IL-8	46	Median: 538.20 pg/ml/min; 95% CI: 189.85, 925.39
IL-8	100	Median: 231.13 pg/ml/min; 95% CI: −39.97, 510.83

### Heavy reliance on ATN breastfeeding associated with evening peak in CRP secretion rate, a pattern exacerbated by concurrent reliance on pumping

**Figure 3:**
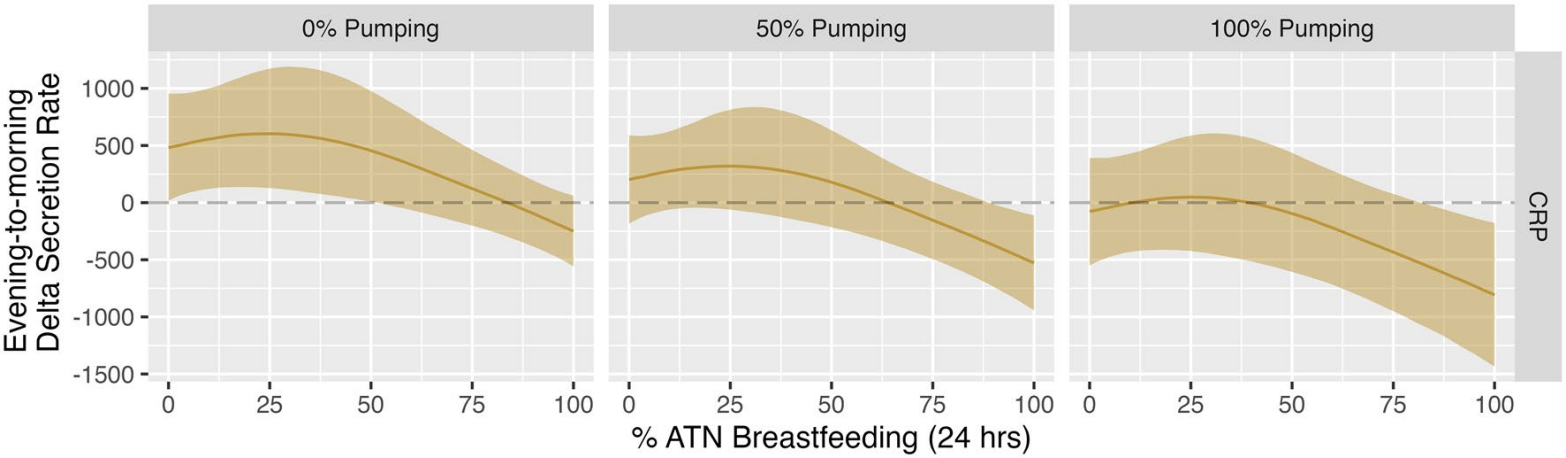
Predicted median evening-to-morning change in CRP by % ATN breastfeeding at 0%, 50% and 100% pumping. Solid lines: point estimates for predicted median value. Shaded regions: 95% credible intervals. Dotted lines: zero difference between evening and morning secretion rate. Values above the horizontal dotted line: secretion rate is higher in the morning. Values below the horizontal dotted line: secretion rate is higher in the evening.

As shown in Table S8, median evening CRP secretion rate was 145.02 pg/mL/min (IQR = 287.09 pg/mL/min) while median morning CRP secretion rate was 164.87 pg/mL/min (IQR = 460.40 pg/mL/min). As shown in Fig. 1 and Table 5, we observed a strong non-linear effect of % ATN breastfeeding on evening-to-morning change in CRP secretion rate (Variance = 411.54; 95% CI = 14.28, 1371.3). Among mothers reporting 0% ATN breastfeeding, estimated evening-to-morning change in CRP secretion rate was positive (median: 377.95 pg/ml/min; 95% CI: −41.86, 801.37), indicating CRP secretion rate peaked in the morning. Among mothers reporting 100% reliance on ATN breastfeeding, estimated evening-to-morning change in CRP secretion rate was negative (median: −352.97 pg/ml/min; 95% CI: −682.15, −24.56), indicating CRP secretion rate peaked in the evening. As Fig. 1 shows, the highest predicted evening-to-morning change in CRP secretion rate (Median: 495.16 pg/ml/min; 95% CI: 75.08, 1026.14) occurred among mothers reporting 25% ATN breastfeeding. We also found a negative linear effect of % pumping (Table S9, Fig. S3). Figure 3 shows the effect of different % pumping values on predicted values for the change (delta) in evening to morning CRP secretion rate.

## Discussion

In our sample of 96 mothers in the Seattle metro area, most women utilized at-the-nipple (ATN) breastfeeding in addition to other infant feeding methods, with pumping as the most common alternative method. Exclusive formula feeding was nonexistent while long-term exclusive ATN breastfeeding (with no history of supplementation) was reported by less than 10% of participants. These results show that, even in a sample of women reporting various markers of high socioeconomic status (e.g., high household income, high educational attainment, and presence of spouse/domestic partner), most women report mixed feeding in the first 6 months postpartum.

We did not observe any robust effects of ATN breastfeeding (or pumping) on evening-to-morning change in IL-1ß, IL-6, or TNF-α secretion rate. We did, however, find a strong non-linear effect of ATN breastfeeding on diurnal IL-8 secretion rate. Predicted evening-to-morning rise in IL-8 was highest among mothers in the later postpartum period who reported using ATN breastfeeding for roughly a quarter of all infant feeding bouts. We also observed a robust non-linear effect of ATN breastfeeding on diurnal CRP, wherein evening-to-morning rise in CRP secretion rate was highest among women who reported using ATN breastfeeding for approximately half of all infant feeding bouts (with no concurrent use of pumping). On a proximate level, the estimated effects of ATN breastfeeding on CRP (a non-specific measure of systemic inflammation) may be at least partially driven by the downstream effects of ATN breastfeeding on IL-8 activity. IL-8 is a strong chemoattractant with high specificity for neutrophils, a type of granulocyte that induces inflammatory cascades^14,48–50^. Neutrophils may therefore act as key mediators between changes in IL-8 and CRP secretion rates. We strongly recommend that subsequent research investigate the prevalence and activation status of circulating neutrophils among postpartum women practicing different infant feeding methods.

Given that morning rise in IL-8 and CRP secretion rates were highest among women who reported using ATN breastfeeding for less than half of all infant feeding bouts, our results suggest that “part-time” ATN breastfeeding may induce and/or be a consequence of relatively heightened inflammatory activation, with potential implications for maternal response to infectious disease challenges. Such “part-time” ATN breastfeeding might induce inflammation due to longer intervals between breastfeeding bouts, increased milk stasis, altered hormone production, and less energetic throughput. On the other hand, upstream factors that limit ATN breastfeeding (e.g., psychosocial stress, infant-led weaning, return to work) may drive the association between proportionally less frequent use of ATN breastfeeding and comparatively higher evening-to-morning rise in IL-8 and CRP secretion rates. We attempted to control for as many of these factors as possible, but the cross-sectional nature of this study precludes a definitive statement on causality. In reality, these relationships are probably complex and arrows of effect may go in both directions (e.g., reduced time spent ATN breastfeeding may cause stress about breastfeeding goals, exacerbating existing inflammation).

In addition to our observation that evening-to-morning rise in CRP secretion rate was highest among women who reported moderate rates of ATN breastfeeding, we also found that evening-to-morning change in CRP secretion rate was negative among women who engaged in proportionally more ATN breastfeeding and/or pumping: in this group, CRP secretion rate peaked in the *evening*. The existing literature indicates that, in most sample populations, “normal” diurnal salivary CRP is characterized by a morning peak in CRP, with adverse conditions being associated with a relatively greater increase in morning CRP levels^40,43,44^. Our results therefore suggest that sustained lactation, via either ATN breastfeeding or pumping, induces an entirely unique immunological context that blunts or reverses this “normal” diurnal pattern.

Sleeping through the night is often regarded as the “ideal” version of infant sleep in contemporary Western populations. However, human lactation evolved under the circumstances of on-demand breastfeeding and waking throughout the night (‘breastsleeping’) ^51^, reflecting the highly digestible nature of human milk (compared to alternatives)^52^. It is possible that CRP peaking in the evening (as opposed to the morning) may be due, in part, to a higher number of sleep–wake cycles among women who are regularly breastfeeding at-the-nipple and/or using pumps. Disruption in sleep–wake cycles corresponds to altered cortisol, melatonin, and human growth hormone production. Considering the well-established effects of these hormones on regulating diurnal shifts in immune function^53,54^, changes in the diurnal production of these hormones may mediate the relationship we find between heavy reliance on lactation and diurnal CRP.

It is also possible that on-demand lactation throughout the day enhances early nighttime immune activity (e.g., phagocytosis, wound healing) as an adaptive response, to the direct benefit of the mother. Prolactin, a hormone produced at particularly high levels during regular lactation^16^, has already been shown to induce beneficial pro-inflammatory cascades during slow wave sleep (which occurs during the first few hours of sleep) in non-postpartum populations^55–58^. It is therefore tempting to speculate that regular lactation via either ATN breastfeeding or pumping is directly and uniquely beneficial for nighttime immune recovery. While we controlled for the presence/absence of night feeding, we did not collect detailed data on the frequency, timing, or duration of night feedings. Future research is therefore needed to parse out the effects of altered sleep patterns versus lactation itself on both hormone levels (e.g., cortisol, melatonin) and immune status among mothers who report similar lactation practices but varying sleep duration/quality.

While we believe that the results we present here can serve as a crucial springboard for future research, we acknowledge the limitations. We used data from a relatively homogeneous sample of women in the USA and therefore these results need to be replicated across diverse populations. Additionally, our findings shed light on the first 6 months of the postpartum only and we make no claims regarding long-term effects. Given budgetary and time constraints, our sample was limited to postpartum women; future research would benefit from comparisons with pregnant and regularly menstruating women to further contextualize the results we report here. Lastly, the method we used to measure the absolute and relative frequency of lactation (either at-the-nipple or via pumping) did not consider the specifics of each breast emptying episode (e.g., which breast was used, the intervals between episodes). We therefore recommend that future research include these more fine-grained measures.

Despite these limitations, this study introduces a novel method for measuring the complexities of maternal lactation practices (and infant feeding behavior more broadly), shows that such behavior is far more complex than most existing metrics are able to capture, and provides strong evidence that variation in maternal lactation practices corresponds to robust differences in postpartum maternal immune status. The first step towards understanding variation is exposing it. We therefore hope this study encourages and informs future research mapping variation in maternal lactation practices and postpartum immune status to specific health outcomes and supports continuing efforts to understand and eliminate maternal morbidity and mortality.

## Methods

### Ethics declaration

This study was approved by the Institutional Review Board at the University of California, Santa Barbara [IRB #1-20-0574] and was conducted in accordance with all corresponding guidelines and regulations. Each participant read and signed an online informed consent form, participation in the study was completely voluntary and anonymous (thus ensuring privacy and autonomy), and all participants were compensated accordingly for their time.

### Participants

Our sample was limited to postpartum women between the ages of 25 and 42 who lived in Seattle, WA, USA, had given birth within the last 6 months, and who reported an absence of previous cancer diagnosis, periodontal disease, and regular tobacco use/vaping. All participants (n = 96) completed a single 24-h collection period, beginning at 12 PM on day one and ending at 12 PM on day two. Because data for this study were collected during the height of the COVID-19 pandemic (September 29th, 2020–July 28th, 2021), research protocols were specifically designed to reduce in-person contact. Saliva was selected for its minimally invasive nature, ease of at-home collection, and similarly detectable levels of cytokines compared to plasma^59^. Collection kits were mailed to each participant before the start of the 24-h collection period and all data were collected from home.

### Infant feeding behavior

Participants were asked to keep an infant feeding record noting the method(s) used during each infant feeding bout and the total number of feedings completed during the 24-h time frame. Possible infant feeding methods included use of (1) other liquids, (2) semi-solid/solid foods, (3) pumped/expressed milk given to infant without storing, (4) participant’s own previously refrigerated/frozen pumped/expressed milk given to infant, (5) donated milk, and (6) breastfeeding at-the-nipple. Based on initial participant feedback, pumping/expressing milk *for later use* was added as an option part way through the study. When applicable, participants were able to indicate multiple methods used in a single feeding bout. Our final measure of ATN breastfeeding was calculated as the percentage of total infant feedings occurring via at-the-nipple breastfeeding during the 24-h collection period (% ATN breastfeeding). Pumping was calculated as the percentage of total infant feedings that involved the use of fresh or frozen expressed/pumped milk and any reported instances of pumping/expressing for future use (% pumping). We decided to calculate each infant feeding method as a percentage rather than use absolute frequency in order to account for differences in infant demand, considering our sample included infants ages 0–6 months old and that the number of total feedings in the 24-h period ranged from 5 to 20. Infant feeding behavior reported during the 24-h sampling interval generally corresponded to infant feeding behavior reported for the two weeks preceding data collection (Fig. S1).

### Salivary immune markers

Each participant was asked to collect two passive drool saliva samples during the 24-h collection period. Participants were instructed to collect the first sample before going to bed on day one and the second at the time of waking the following morning. To ensure sample integrity, participants were instructed to place saliva samples in a home freezer immediately after collection. At the end of the 24-h collection period, saliva samples were transported on ice and stored at −80 °C.

Saliva samples were then shipped on dry ice to the Salimetrics SalivaLab (Carlsbad, CA) where they were tested for IL-1β, IL-6, TNF-α, and IL-8 cytokines and C-reactive protein (CRP) (Table 1). The cytokine panel was performed using a proprietary electrochemiluminesence method developed and validated for saliva by Salimetrics, while the CRP assay was performed using a high sensitivity enzyme immunoassay (Cat. No. 1-2102: https://salimetrics.com/wp-content/uploads/2017/05/c-reactive-protein-saliva-elisa-kit.pdf). All tubes were weighed before analysis, all assays were run in duplicate, and the resulting mean values were used in all final analyses. Samples with initial values greater than the maximum value of the standard curve were diluted and rerun for accurate results according to manufacturer guidelines. For the cytokine panel, the average coefficient of variation for all samples tested was < 15%, which meets Salimetric’s criteria for accuracy and repeatability in Salivary Bioscience and exceeds the applicable NIH guidelines for Enhancing Reproducibility through Rigor and Transparency. For the CRP assay, the average intra-assay coefficient of variation was 3.20% and the average inter-assay coefficient of variation was 2.61%, which meets the manufacturers’ criteria for accuracy and repeatability in Salivary Bioscience and exceeds the applicable NIH guidelines for Enhancing Reproducibility through Rigor and Transparency. For assay sensitivity and standard curve range values, see Table S1.

To account for the effects of salivary flow rate, absolute concentrations of all salivary biomarkers were transformed into secretion rates before analysis. For each saliva sample, there was a recorded duration (in seconds) that it took for the participant to collect the sample as well as a corresponding weight (in grams) of the final sample. Since saliva is approximately 99% water, it is standard practice to use a 1:1 conversion rate for grams to milliliters^60^. We therefore converted each sample weight to milliliters and the duration into minutes and calculated the flow rate (mL/min) for each sample. Salivary flow rate did not vary by infant feeding method (% ATN breastfeeding or % pumping) or sampling time (bedtime versus waking) (Fig. S2). Secretion rate of each analyte for each sample was calculated as the raw concentration (unit/mL) multiplied by the flow rate (mL/min).

To account for individual variation in baseline immune status^61^ and better quantify the effects of ATN breastfeeding on immune response patterns, we used absolute morning and evening salivary cytokine secretion rates to calculate each participant’s evening-to-morning change (i.e., delta) in secretion rate. Delta secretion rate, which we used as the outcome variable in all models described below, was calculated by subtracting evening secretion rate from morning secretion rate: positive delta values indicated that secretion rate was higher in the morning, negative delta values indicated secretion rate was lower in the morning, and a delta value of zero indicated no difference between evening and morning secretion rate. Due to issues with several saliva samples (e.g., outside detection threshold), evening-to-morning change in CRP secretion rate was successfully calculated for 92 out of 96 participants.

### Statistical analyses

All analyses were executed in R 4.3.1 (https://cran.r-project.org). Using the *brms* package^62^, we employed Bayesian regression models to estimate the effects of % ATN breastfeeding on evening-to-morning change in IL-1β, IL-6, IL-8, TNF-α, and CRP secretion rate. To explicitly separate the effects of ATN breastfeeding from pumping, all models accounted for the effects of % pumping. All models also accounted for the fixed effects of days since delivery, time between saliva sample collections, maternal age, pre-pregnancy BMI, income, educational attainment, delivery mode (C-section versus vaginal birth), presence/absence of reported pregnancy complications, and presence/absence of co-sleeping, night feeding, and alcohol consumption during the 24-h collection period. We also controlled for whether a participant completed the study before or after the addition of “pumping/expressing milk for later use” as an infant feeding method. Lastly, all models accounted for “pandemic score”, an integer variable created by summing the number of adverse effects reported as a result of the COVID-19 pandemic (i.e., loss of employment, financial stress, loss of childcare, heightened anxiety, social isolation, reduced access to postpartum support, and reduced access to mental health services). These data were collected via a “Select all that apply” survey question designed by the researchers to approximate the perceived effects of the rapid societal changes induced by the COVID-19 pandemic and associated stay-at-home order. Finally, all categorical covariates were converted into dummy numerical variables before modeling to allow for standardization of posterior predicted values.

Because the effects of % ATN breastfeeding on CRP and IL-8 were non-linear (as determined by comparing variance parameter values to linear coefficients), spline regression model formulas were used for these outcome variables. All other outcome variables were modeled using linear regression formulas. All models were run using two chains for between 10,000 and 25,000 iterations and used loose priors and Gaussian distributions. All models had R-hat values of 1, indicating appropriate chain mixing and convergence. Predicted values were standardized by the mean value for % pumping (18.71), median values for all continuous covariates (Table 2), and middle values for all dummy categorical covariates (e.g., 0.5 for a binary dummy variable) (Table 3).

### Supplementary Information

Supplementary Information.

## Data Availability

Data and R code for the statistical analyses are published on GitHub (https://github.com/carmenhove/sphs)^63^.
