# Supplementary material for: Variation in maternal lactation practices associated with changes in diurnal maternal inflammation
Source: Sci Rep. 2024 Feb 22;14:4376. doi: 10.1038/s41598-024-54963-4 (PMC10883910; doi:10.1038/s41598-024-54963-4)
Supplement: Supplementary file 1 — Supplementary Information. [file 41598_2024_54963_MOESM1_ESM.pdf]

Supplementary Information

Title: Variation in maternal lactation practices associated with changes in diurnal maternal inflammation

Authors

Author: Carmen Hove*
Email: carmenhove@ucsb.edu
Affiliation: University of California, Santa Barbara

Author: Kristine Joy Chua
Email: kristinechua@ucsb.edu
Affiliation: University of California, Santa Barbara

Author: Melanie Ann Martin
Email: martinm7@uw.edu
Affiliation: University of Washington

Author: Madison Hubble
Email: hubblem@uw.edu
Affiliation: University of Washington

Author: Amy M Boddy*
Email: boddy@anth.ucsb.edu
Affiliation: University of California, Santa Barbara

Supplementary Tables

Table S1. Assay sensitivity, range, and coefficient of variability by salivary measure.

Measure	Sensitivity	Range	Coefficient of Variability
CRP	0.042 pg/mL	25-1600 pg/mL	2.61%
IL-6	0.0491 pg/mL	0.0491-736 pg/mL	5.00%
IL-1ß	0.0195 pg/mL	0.0195-589 pg/mL	2.51%
IL-8	0.0201 pg/mL	0.0201-574 pg/mL	3.80%
TNF-α	0.0314 pg/mL	0.0314-380 pg/mL	6.46%

Table S2. Sample breakdown by household income bracket.

Income	N	Percent
$20,000 - $39,999	4	4.17%
$60,000 - $79,999	5	5.21%
$80,000 - $99,999	7	7.29%
$40,000 - $59,999	8	8.33%
More than $100,000	72	75.00%

Table S3. Sample breakdown by maternal educational attainment.

Education	N	Percent
High school graduate	5	5.21%
College degree	38	39.58%
Professional degree	53	55.21%

Table S4. Sample breakdown by maternal relationship status.

Relationship Status	N	Percent
Single	2	2.08%
Domestic Partnership	8	8.33%
Married	86	89.58%

Table S5. Sample breakdown by maternal race/ethnicity.

Race/Ethnicity	N	Percent
Black/African American	1	1.04%

Race/Ethnicity	N	Percent
Native American/Alaska Native	1	1.04%
Hispanic	3	3.12%
Asian	10	10.42%
Multi-racial/multi-ethnic	11	11.46%
European	65	67.71%

Table S6. Sample breakdown by maternal employment status.

Employment Status	N	Percent
Student	2	2.08%
Unemployed looking for work	3	3.12%
Employed part time	9	9.38%
Other (please specify)	9	9.38%
On un-paid maternity leave	10	10.42%
Unemployed not looking for work	11	11.46%
Employed full time	15	15.62%
On paid maternity leave	37	38.54%

Table S7. Incidence rate of infant feeding behaviors.

Infant Feeding Behavior	Yes	No	Incidence
Ever donated	3	93	3.12%
Exclusive ATN breastfeeding	8	88	8.33%
No ATN breastfeeding (24 hr)	11	85	11.46%
Ever used solid food	15	81	15.62%
Ever used non-breastmilk liquids	31	65	32.29%
Exclusive ATN breastfeeding (24 hr)	37	59	38.54%
Mixed feeding (24 hr)	48	48	50%
Ever pumped	82	14	85.42%
Ever ATN breastfed	94	2	97.92%

Table S8. Descriptive statistics for absolute CRP and cytokine values by sample (1 = evening, 2 = morning).

Measure	Sample	Unit	Median	IQR	Mean	Standard Deviation	Min	Max
CRP	1	pg/mL/min	145.02	287.09	364.93	618.22	9.22	4511.32

Measure	Sample	Unit	Median	IQR	Mean	Standard Deviation	Min	Max
CRP	2	pg/mL/min	164.87	460.40	439.36	717.56	8.29	5163.67
IL-1ß	1	pg/mL/min	16.89	32.39	52.41	157.27	1.30	1473.73
IL-1ß	2	pg/mL/min	67.06	145.73	275.41	738.02	2.77	5106.43
IL-6	1	pg/mL/min	1.98	2.59	3.24	4.80	0.20	41.59
IL-6	2	pg/mL/min	1.37	2.80	4.08	10.50	0.15	79.27
IL-8	1	pg/mL/min	127.79	216.83	263.29	625.60	9.92	5566.19
IL-8	2	pg/mL/min	269.24	457.38	562.01	853.16	19.19	5221.01
TNF-α	1	pg/mL/min	1.00	1.61	1.75	2.54	0.10	17.88
TNF-α	2	pg/mL/min	1.54	4.03	4.63	9.45	0.14	73.15

Table S9. Model coefficients and associated 95% credible intervals for the estimated effects of % ATN breastfeeding, % pumping, and days since delivery.

Measure	Parameter	Coefficient
IL-1ß	% ATN breastfeeding	ß = 0.45 pg/ml/min/%; 95% CI = -4.18, 5.03
IL-6	% ATN breastfeeding	ß = 0 pg/ml/min/%; 95% CI = -0.05, 0.06
TNF-α	% ATN breastfeeding	ß = 0 pg/ml/min/%; 95% CI = -0.06, 0.05
CRP	% pumping	ß = -5.6 pg/ml/min/%; 95% CI = -10.93, -0.21
IL-1ß	% pumping	ß = -3.37 pg/ml/min/%; 95% CI = -8.69, 2.01
IL-6	% pumping	ß = -0.04 pg/ml/min/%; 95% CI = -0.1, 0.03
IL-8	% pumping	ß = 0.07 pg/ml/min/%; 95% CI = -4.47, 4.62
TNF-α	% pumping	ß = -0.02 pg/ml/min/%; 95% CI = -0.08, 0.05
CRP	Days since delivery	ß = 0.06 pg/ml/min/day; 95% CI = -2.29, 2.44
IL-1ß	Days since delivery	ß = -0.4 pg/ml/min/day; 95% CI = -2.62, 1.82
IL-6	Days since delivery	ß = -0.01 pg/ml/min/day; 95% CI = -0.03, 0.02
IL-8	Days since delivery	ß = 2.15 pg/ml/min/day; 95% CI = 0.19, 4.1
TNF-α	Days since delivery	ß = 0.01 pg/ml/min/day; 95% CI = -0.02, 0.04

Supplementary Figures

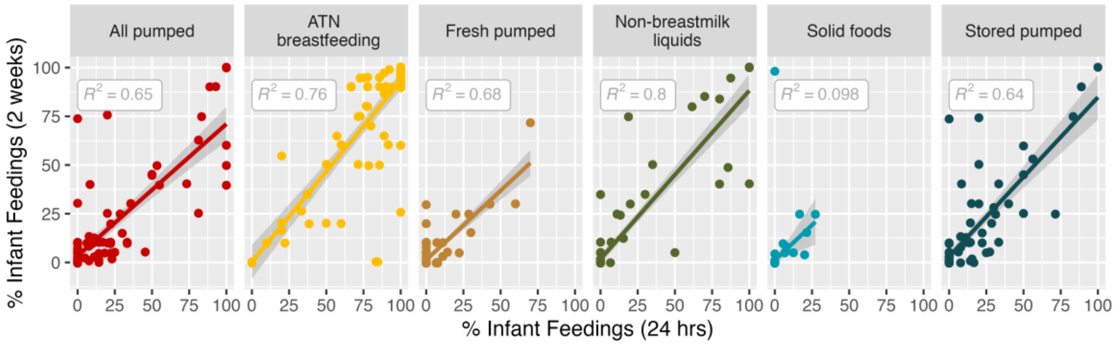

Fig S1. Correlation between reported reliance on infant feeding method during the 24-hour collection period versus the prior 2 weeks. While participants were able to indicate multiple methods for each feeding bout in the 24-hour colleciton period, the question regarding the preceding two weeks was phrased differently and required each percentage to add up to 100%.

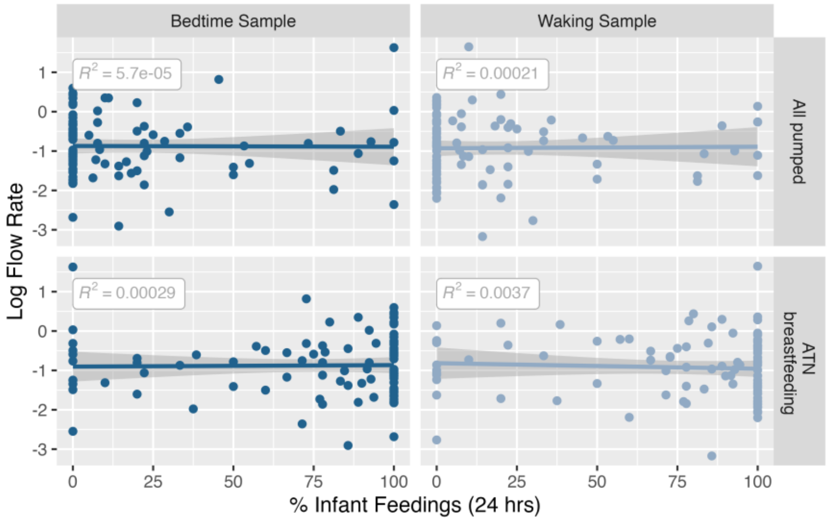

Fig S2. Log flow rate by % ATN breastfeeding and % pumping by sample (bedtime versus waking).

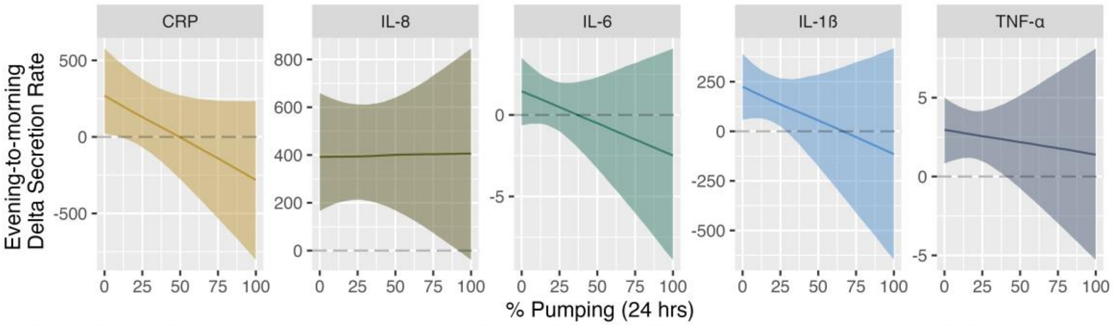

Fig S3. Predicted median evening-to-morning change in CRP, IL-8, IL-6, IL-1ß, and TNF-α secretion rates by % pumping. Solid lines = point estimates for predicted median value. Shaded regions = 95% credible intervals. Dotted lines = zero difference between evening and morning secretion rate. Values above the horizontal dotted line = secretion rate is higher in the morning. Values below the horizontal dotted line = secretion rate is higher in the evening.

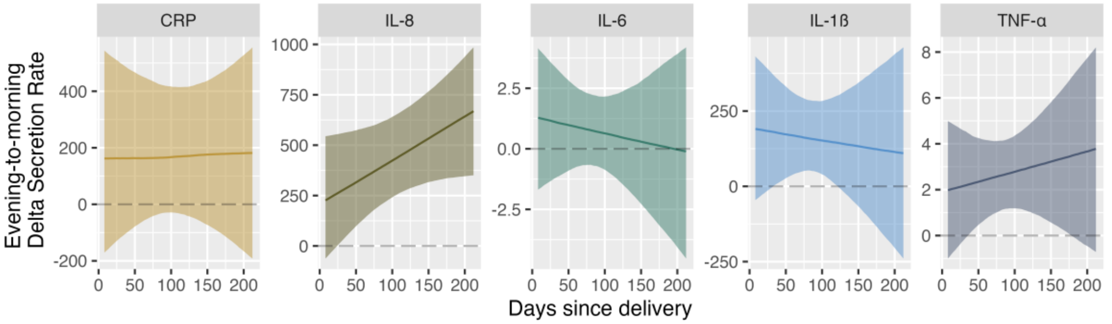

Fig S4. Predicted median evening-to-morning change in CRP, IL-8, IL-6, IL-1ß, and TNF-α secretion rates by days since delivery. Solid lines = point estimates for predicted median value. Shaded regions = 95% credible intervals. Dotted lines = zero difference between evening and morning secretion rate. Values above the horizontal dotted line = secretion rate is higher in the morning. Values below the horizontal dotted line = secretion rate is higher in the evening.
